# Remedies as Time-Keepers

**Published:** 1885-11

**Authors:** C. Carleton Smith

**Affiliations:** Philadelphia


### REMEDIES AS TIME-KEEPERS.

There are remedies incorporated in our *Materia Medica* which may justly be termed "Time-keepers," from the fact that in their provings they elicit symptoms which re-occur at certain hours or at stated periods, and at certain and regular intervals, without fail.

This feature, belonging to certain drugs, being verified by each and every prover, enables us to prescribe them with the utmost confidence when indicated—this confidence being daily strengthened by the witnessing of most remarkable cures following their administration.

One of the most important of this class of drugs, and the one I wish to call attention to particularly in this brief paper, is the Aranea-diadema, commonly called the cross-spider. When the proving of this drug was first made, one of the most peculiar symptoms which was prominently brought forth and verified by subsequent provers is the one which reads: "Toothache every day at the same hour." Now, although this symptom is exactly in accordance with the proving, yet we must not make the mistake, as is frequently done by those who do not properly interpret the true meaning of symptoms as recorded in our *Materia Medica*, to throw aside this drug in a given case of toothache, for the reason that it may not happen to occur in the patient under treatment at precisely the same hour each day—remembering, that it not so much the precise hour at which the symptom returns, as it is the marked regularity.

Many brilliant cures have been lost to the practitioner, and many a convert, also, to our school of practice, by reason of this error.

In the homœopathic use of such drugs as the one we are now considering, we must reason from analogy, and if the symptom, whether it be odontalgia or what not, comes on with clock-like regularity every day, every other day, or every seventh day, moving as it were in a cycle, then the remedy, corresponding to this positive regularity, is homœopathic to it, and it will, in all likelihood, effect a cure.

By thus reasoning I was enabled to make a most beautiful and perfect cure of a case of chills in the person of a robust, tall, and heavily bearded man, who, from the nature of his business, was exposed to all sorts of weather.

This patient came to my office last spring, and explained to me that he was suffering from a very peculiar ailment. For weeks past, though feeling tolerably well throughout the day, attending regularly to his daily round of duties, every night, as soon as he stepped into bed, he was seized with a severe chill, which lasted perhaps for the space of two hours, followed by heat, but no sweat.

Thinking that the cold sheets upon which he lay had something to do with his difficulty, he adopted the plan of substituting blankets, but no improvement followed this procedure. The key-note in the case, "chill the moment he touches the bed," is very remarkable, and I did not propose wasting half a day hunting for it in the *Materia Medica*, for I knew it was not to be found there. I, however, reasoned from analogy, as I have always been in the habit of doing in prescribing in similar cases, thereby interpreting drug symptoms far beyond their literal meaning, as printed in black and white in our books. Hence, I was enabled to cure the patient promptly and effectually, and in this way. Upon hearing the story of the gentleman's sufferings I said to myself, There is no such symptom to be found, *verbatim et literatim*, among our key-notes; but in a moment the drug Aranea-diadema loomed up before me, with that remarkable symptom confronting me, viz.: "toothache every day at the same hour." Now this patient's sufferings did not present them selves at the same hour precisely each day, but, on the contrary, were developed only when he retired to bed, which was at irregular hours; but when they did present themselves they were as regular as clock-work, and always under the same conditions.

Aranea occurring to me, as I remarked before, as being the best time-keeper among our remedial agents, I was satisfied that this, and this only, afforded hope for my patient. Accordingly, the remedy in the thirtieth potency was administered in smallest size pellets, a dose night and morning for four days.

At the expiration of a week the patient, according to agreement, reported, and stated that after the fourth dose the chills ceased, and he considered himself well. I saw him a week later and there was no relapse.

*Comments.*—We are prone to put a too narrow or contracted interpretation on many of our provings, not allowing them sufficient scope. For instance, Lac-caninum has sore throat, constantly changing from one side to the other. Now, if we stop here, and simply infer that this drug has this peculiar power or action only in the throat, we will do nothing else with this peculiar symptom but cure sore throats with it. If, however, on the other hand, we interpret the symptoms recorded in our provings aright, giving them proper and reasonable construction, and reading between the lines, we will soon find that just as well certain forms of colic or acute pains in various portions of the body can also be readily cured with it, wherever this peculiar symptom exists of the pain alternating from one side to the other at regular intervals. Studying remedies in this light will give us much greater latitude as healers of the sick, and will help us out with many a knotty case, where the identical symptom, as given by the patient, cannot be found recorded.

*Remarks.*—The headaches of Aranea are very severe, greatly aggravated by attempting to read or to write, but ameliorated by smoking tobacco, and entirely relieved by smoking out in the fresh air.

The colic produced by this remedy is somewhat similar in its severity to Colocynth, but while the colic of the latter is much relieved by pressing the abdomen against some hard substance or bending double, the Aranea colic is made better by sitting up and rubbing the abdomen all over with the hand.

The Aranea toothache occurs in one or more teeth every day at precisely the same hour, but on lying down every tooth in the head is sure to ache.

The menstrual symptoms closely resemble Calcarea carb., inasmuch as it causes the flow to appear eight days too soon, and are too copious and too strong.

We have but a meagre proving of Aranea-diadema, and we should be pleased to hear through the pages of THE HOMŒOPATHIC PHYSICIAN of cures made with this drug, with the symptoms clearly given, by members of the profession who have found it, as the writer has, a most valuable addition to our armamentarium.

C. CARLETON SMITH, M. D.

PHILADELPHIA.

